# Bioactive compounds of *Curvularia* species as a source of various biological activities and biotechnological applications

**DOI:** 10.3389/fmicb.2022.1069095

**Published:** 2022-12-08

**Authors:** Tushar Mehta, Mukesh Meena, Adhishree Nagda

**Affiliations:** Laboratory of Phytopathology and Microbial Biotechnology, Department of Botany, Mohanlal Sukhadia University, Udaipur, Rajasthan, India

**Keywords:** *Curvularia* species, secondary metabolites, anti-microbial, biological activities, mycoherbicide, myconanosynthesis, biotechnological applications

## Abstract

Many filamentous fungi are known to produce several secondary metabolites or bioactive compounds during their growth and reproduction with sort of various biological activities. Genus *Curvularia* (Pleosporaceae) is a dematiaceous filamentous fungus that exhibits a facultative pathogenic and endophytic lifestyle. It contains ~213 species among which *Curvularia lunata, C. geniculata, C. clavata, C. pallescens*, and *C. andropogonis* are well-known. Among them, *C. lunata* is a major pathogenic species of various economical important crops especially cereals of tropical regions while other species like *C. geniculata* is of endophytic nature with numerous bioactive compounds. *Curvularia* species contain several diverse groups of secondary metabolites including alkaloids, terpenes, polyketides, and quinones. Which possess various biological activities including anti-cancer, anti-inflammatory, anti-microbial, anti-oxidant, and phytotoxicity. Several genes and gene factors are involved to carry and regulate the expression of these activities which are influenced by environmental signals. Some species of *Curvularia* also show negative impacts on humans and animals. Apart from their negative effects, there are some beneficial implications like production of enzymes of industrial value, bioherbicides, and source of nanoparticles is reported. Many researchers are working on these aspects all over the world but there is no review in literature which provides significant understanding about these all aspects. Thus, this review will provide significant information about secondary metabolic diversity, their biological activities and biotechnological implications of *Curvularia* species.

## Introduction

Fungi are a miscellaneous group of organisms comprising several beneficial and damaging biological activities which have a major impact on flora and fauna of the biome. For example, fungi are known to produce some potent antibiotics such as penicillin and cephalosporin. On other hand, some fungal species are able to synthesize and excrete extremely toxic metabolites, such as aflatoxins, curvularin, helminthosporin, etc. (Timberlake and Marshall, 1989). These filamentous fungi show huge diversity in their metabolites including polyketides, alkaloids, quinones, etc. The metabolic diversity of fungal genera is extraordinary, making it possible for them to perform various biological activities such as anti-oxidant, anti-cancer, anti-viral, anti-microbial, and anti-inflammatory (Khiralla et al., 2019). The genus *Curvularia* Boedijn 1933 (Family: Pleosporaceae; Order: Pleosporales; Class: Dothideomycetes; Phylum: Ascomycota) is a dematiaceous hyphomycete fungus having endophytic and pathogenic nature. Species of this genus are associated with several plants and humans as severe pathogens (Hyde et al., 2014). Species of *Curvularia* are known to synthesize various bioactive compounds including curvulamine, curindolizine, curvupallides, etc. which show anti-microbial and anti-inflammatory activities. Some metabolites like apralactone A, (+)-(15R)-10,11-E-dehydrocurvularin, (+)-(15R)-12-hydroxy-10,11-E-dehydrocurvularin (+)-(11R,15R)-11-hydroxycurvularin (+)-(15R)-12-oxocurvularin have cytotoxic activity, and shows toxic effects on host (Greve et al., 2008a; Han et al., 2014). Due to the secretion of such types of compounds with vital activities, different strains of *Curvularia* are a useful tool in the field of biotechnology nowadays. With the help of these fungi, several processes are ongoing in this field like production of enzymes of industrial value, use as a tool for biotransformation of the host cells to increase desirable products, to remove heavy metal toxicity from contaminated sites, for production of substances of economic values such as biofuels, biosorbents, bioemulsifiers, and so many others (Bengyella et al., 2019). Several other microorganisms like actinomycetes (e.g., *Streptomyces* sp.), bacteria (e.g., *Pseudomonas, Pedobacter, Bacillus* sp.) also have capacity to produce valuable metabolites but filamentous fungi are superior in secretory capacity having diverse bioactive metabolic molecules and are beneficial in fermentation systems (Meyer et al., 2016; Rani et al., 2021). Thus, the use of endophytic fungal genera can fill gaps in the production of compounds of biotechnological and economic value. So, in the current scenario, pollution and environmental crisis are the major problems. The application of filamentous fungi and their bioactive compounds can be a good source of ecofriendly biotechnological processes. The present article deals with various bioactive secondary metabolites of different species of *Curvularia*, their biological activities and their application in the field of biotechnology for the production of valuable compounds. The review article will provide a better understanding and applicable knowledge about bioactive compounds (metabolites) of the genus *Curvularia*.

## Diversity of secondary metabolites of *Curvularia* species

Secondary metabolites (SMs) are low molecular weight organic compounds which do not participate directly in plant growth and development but involved in signaling and regulation of primary metabolic pathways (Kroymann, 2011). They also play significant roles in response to changing environment, growth, and development (Berini et al., 2018). There are various diverse groups of secondary metabolites are known which are secreted by plants and microorganisms. Among all microorganisms, fungal groups play a pivotal role in secondary metabolites production (Chutulo and Chalannavar, 2018). These include biological activities like anti-oxidant, anti-bacterial, anti-malarial, anti-biofouling, anti-larval, anti-inflammatory, anti-fungal, anti-cancer, leishmanicidal, and phytotoxicity. *Curvularia* sp. obtained from *Rauwolfia macrophylla* produced some bioactive secondary metabolites like 2'-deoxyribolactone, hexylitaconic acid, and ergosterol which have anti-microbial, anti-oxidant, and acetylcholinesterase inhibition activities (Kaaniche et al., 2019). A new flavanoid epicatechin (DoE) has been extracted from *Curvularia australiensis* FC2AP, which display potent anti-cervical cancer activity by inhibiting angiogenesis (Mani et al., 2021). It is also observed that crude extract of *Curvularia lunata*, an endophyte of *Elaeis guineensis* shows potent anti-microbial activity against *Staphylococcus aureus, Candida albicans* and *Pseudomonas aeruginosa* with inhibition zones of 8 ± 0.6, 10 ± 1.5, and 2 ± 1.0 mm, respectively. Futher, GC-MS analysis confirmed the presence of 2,4-di-tert-butylphenol, heptadecane, 2,6,10,14-tetramethyl, tetradecanoic acid, 2-hydroxy-, methyl ester, γ-terpinene, p-cymene, and oxirane (chloromethyl) in the crude extract of the fungus (Nwobodo et al., 2022). An *in vitro* study was done by Kalimuthu et al. (2022) that conclude the anti-oxidant and anti-cancer potential of *Curvularia geniculata* isolated from *Phyllanthus niruri* L. They extract total 13 compounds from which 2-methyl-7-phenylindole shows high binding affinity for epidermal growth factor receptor (EGFR), having high radical scavenging activity and cytotoxicity against HepG2 cell lines with IC_50_ value of 62.23 μg/mL. *Curvularia* species produce a vast diversity of secondary metabolites in which polyketides, alkaloids, quinones, and terpenes are major groups.

### Polyketides

Polyketides are a structurally diverse group of biologically active secondary metabolites that derived from natural sources such as plants, fungi, bacteria and animals (McDaniel et al., 1993). Polyketides are synthesized by multi-domain enzymes polyketide synthases (PKSs) (Curran et al., 2018). A study done by Lu et al. (2022) demonstrated that polyketides synthase gene *Clpks18* play a vital role in pathogenicity of *Curvularia lunata* by influencing its metabolic network. It is also observed that deletion of this gene reduces the production of the toxin methyl 5-(hydroxymethyl) furan-2-carboxylate which also reduces the virulence of fungus. There are 46 polyketides isolated from *Curvularia* species which have phytotoxicity, cytotoxicity, anti-oxidant, anti-microbial, and anti-larval activities (Khiralla et al., 2019). 11-Methoxycurvularin and (*S*)-5-ethyl-8, 8-dimethylnonanal isolated from *Curvularia oryzae* MTCC 2605 which shows cytotoxic activities against fungi, bacteria and 4th instar *Spodoptera litura* larvae (Busi et al., 2009). *Curvularia* sp. strain M12 is an endophyte of *Murraya Koenigii* possesses several metabolites in which murranofuran A, murranolide A, murranopyrone, murranoic acid A, N-(2-hydroxy-6-methoxyphenyl) acetamide curvularin, (S)-dehydrocurvularin, pyrenolide A, modiolide A, and 8-hydroxy-6-methoxy-3-methylisocoumarin are major components. Among these metabolites, pyrenolide A shows potent motility impairing activity against zoospores of *Phytophthora capsici* (Mondol et al., 2017). Five new hybrid peptide-polyketides, curvularides A-E isolated from the endophytic fungus *Curvularia geniculata* in which curvularide B possesses anti-fungal activity against *Candida albicans* (Chomcheon et al., 2010).

**Figure 1:**
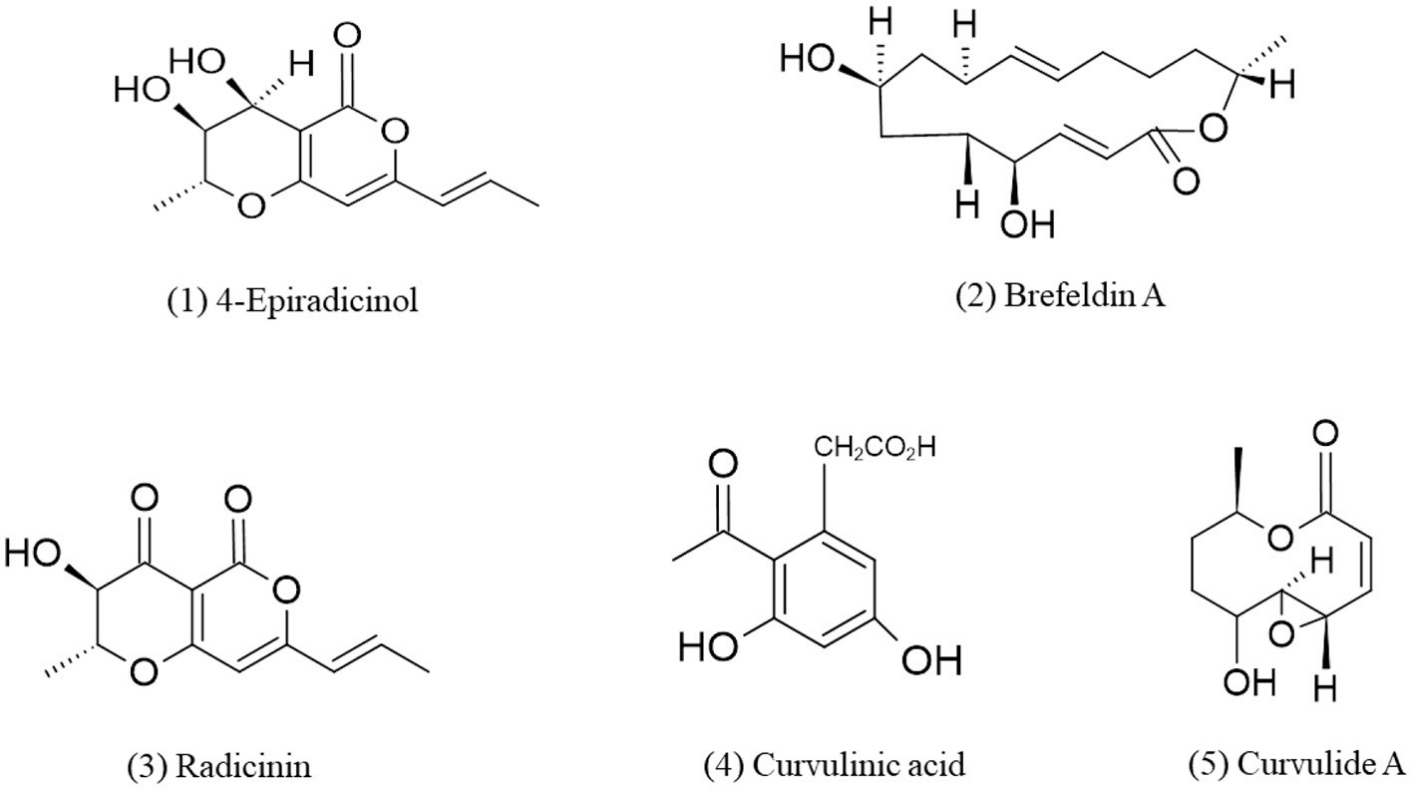
Chemical structures of bioactive compounds of polyketides family of *Curvularia* spp. (1) 4-Epiradicinol, (2) Brefeldin A, (3) Radicinin, (4) Curvulinic acid, and (5) Curvulide A.

Gorst-Allman et al. (1982), earlier reported metabolite *7-epi-* Brefeldin A from *Curvularia lunata*. *Curvularia* sp. isolated from marine red algae *Acanthophora spicifera* produces macrolide apralactone A and some curvularin macrolides which shows cytotoxic activity against human tumor cell lines (Greve et al., 2008b). Ten membered lactones are also isolated by marine-derived *Curvularia* sp. NMR spectra revealed that these lactones closely resembled pyrenolide A (Greve et al., 2008b). Three new metabolites, curvulapyrone, curvulalide, and curvulalic acid together with six known compounds, modiolides A and B, pyrenolide A, stagonolide E, mycoepoxydiene, and deacetylmycoepoxydiene have been reported from the sea fan-derived fungus *Curvularia* sp. PSUF22 by chromatographic techniques and metabolites were identified by spectroscopic techniques such as UV, IR, NMR, MS, etc. (Trisuwan et al., 2011). Phenylacetic acid derivatives, methyl 2-acetyl-3,5-dihydroxyphenylacetate (1) and methyl 2-acetyl-5-hydroxy-3-methoxyphenylacetate (2) curvulin (3) or ethyl 2-acetyl-3,5-dihydroxyphenylacetate (4), a known metabolite of *Curvularia siddiquii*, and 4-epiradicinol (5) have been isolated from the culture mycelia of *Curvularia lunata* in which compound 5, 4 epiradicinol exhibit anti-microbial activity against *Staphylococcus aureus, Escherichia coli, Bacillus subtilis*, and *Salmonella choleraesuis* whereas compound 1, 3, and 4 lacks anti-microbial and anti-oxidant activity (Varma et al., 2006). Phthalic acid butyl isobutyl ester and radicinin which are potent phytotoxins have been isolated from the culture of *Curvularia* sp. FH01 which found inside the gut of *Atractomorpha sinensis* and shows anti-fungal and herbicidal activities (Zhang et al., 2011). The first report of the bioactive polyketides from endolichenic fungus *Curvularia trifolii* which is an endophyte of *Usnea* sp. has been given by Samanthi et al. (2015). They isolated two new compounds and identified as macrocyclic lactone (compound 1) and macrocyclic ketone (compound 2) using techniques such as 1H, 13C NMR, DEPT, HMBC, HSQC, FABMS, etc. these compounds show potent anti-cancer, anti-inflammatory and anti-oxidant activities *via* the radical scavenging activity with IC_50_ values of 4.0 ± 2.6 and 1.3 ± 0.2 mg/mL by compound 1 and 2, respectively. Compound 2 shows higher radical scavenging activity than compound 1. Curvulaide A which is a bicyclic polyketide has been obtained from marine-derived fungus *Curvularia* sp. IFB-Z10 and identified by data of 1D and 2D nuclear magnetic resonance (NMR), electronic circular dichroism (ECD) spectra and by high-resolution electrospray ionization mass spectrometry (HRESIMS) (Liu et al., 2019). This compound has potent anti-anaerobic bacterial activity against *Porphyromonas gingivalis* which is a periodontal pathogen (Liu et al., 2019). In a similar strain of *Curvularia*, another bicyclic polyketide named spirocurvulaide has been detected by An et al. (2019). Asperpentyn, a potent polyketide that shows cytotoxicity against Ehrlich ascites carcinoma in mice has been obtained from marine fungus *Curvularia inaequalis* (Smetanina et al., 2014). Some cytochalasin derivatives verruculoid A (dimer), 12-nor-cytochalasin F, 22-methoxycytochalasin B6, 19-hydroxycytochalasin B, and 20-deoxycytochalasin B has been isolated from *Curvularia verruculosa* CS-129, an endozoic fungus of *Shinkaia crosnieri* which is a deep-sea squat lobster. Among which, verruculoid A shows anti-microbial activity against *Escherichia coli* (MIC = 2 μg/mL) while 19-hydroxycytochalasin B display cytotoxic activity against three tumor cell lines (HCT-116, HepG-2, and MCF-7) with IC_50_ values ranges from 5.2 to 12 μM (Hu et al., 2021). The chemical structures of polyketides derived from *Curvularia* spp. are given in Figure 1.

### Terpenes

**Figure 2:**
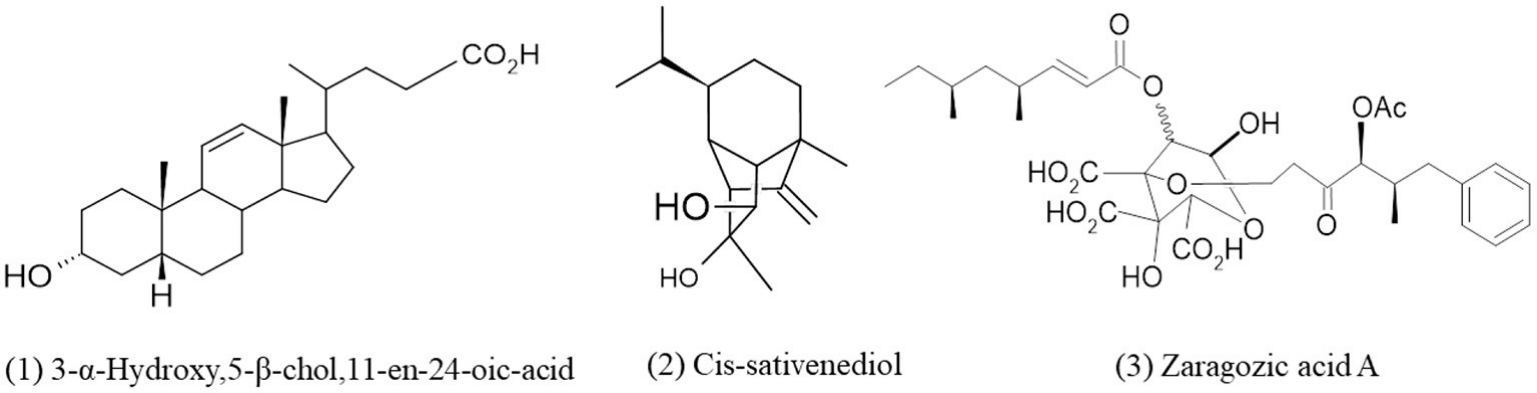
Chemical structures of bioactive compounds of terpenes family of *Curvularia* spp. (1) 3-α-Hydroxy,5-β-chol,11-en-24-oic-acid, (2) Cis-sativenediol, and (3) Zaragozic acid A.

Terpenes or terpenoids are natural chemical compounds built from isoprene units and produced by plants and some other organisms (Sahu et al., 2020). Terpenes are available in large amounts and are the largest group of secondary metabolites with a wide variety of structures. There are numerous types of terpenes, which are based on the number of isoprene units (in brackets) in their structure, such as monoterpenes, generated from geranyle pyrophosphate (2), sesquiterpenes, generated from farnesyl pyrophosphate (3), diterpenes, generated from geranylgeranyl pyrophosphate (4), polyterpenes (n), etc. (Sahu et al., 2020). The main enzymes for biosynthesis of terpenes are terpene synthases (TPSs) which had been described only in fungi and plants in the case of the eukaryotic domain (Chen et al., 2016). Terpenes are copious in marine-derived fungi with well-defined and unique chemical structures having specific biological activities (Elissawy et al., 2015). There are five terpenes known isolated from *Curvularia* species (Khiralla et al., 2019). Bills et al. (1994) isolated *Curvularia lunata* var. *lunata* and *Curvularia lunata* var. *aeria* from the bark of *Ficus elastica* which produced Zaragozic acid A (squalestatins) which is the potent inhibitor of cholesterol biosynthetic enzymes like squalene synthase. (Calam and Robinson, unpublished work) isolated curvularin and dehydrocurvularin from a *Curvularia* species, they obtained a small amount of a crystalline metabolite named 3α-Hydroxy-5P-choZ-11-en-24-oic acid after that Munro et al. (1974) established the compound as bile acid derivative (Figure 2).

### Quinones

**Figure 3:**
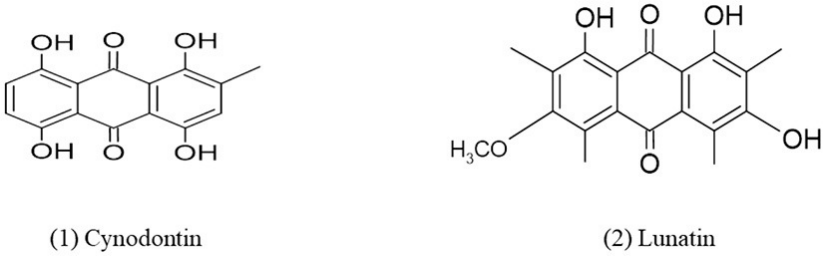
Chemical structures of bioactive compounds of quinone family of *Curvularia* spp. (1) Cynodontin, and (2) Lunatin.

Quinones are a ubiquitous class of compounds that are commonly found in various natural products and synthesized through hydroquinones and/or catechols metabolism (Chhour et al., 2021). Quinones also act as toxicological intermediates which have many hazardous effects including acute cytotoxicity, carcinogenesis, and immunotoxicity (Bolton et al., 2000). The mechanisms by which quinones cause these effects can be quite complex. In 1977, Van Eijk and Roeymans (1977) identified red tetrahydroxyanthraquinone as cynodontin (1,4,5,8-tetrahydroxy-2-methylanthraquinone) 135 from *Curvularia lunata* NRRL 2380 which were isolated earlier. Jadulco et al. (2002), identified two new anthraquinone, 1,3,8-trihydroxy-6-methoxyanthraquinone, later named lunatin and modified bisanthraquinone cytoskyrin A, derived from Fungus *Curvularia lunata* isolated from *Niphates olemda*, a marine sponge. This anthraquinone shows anti-microbial activity against *Staphylococcus aureus, Bacillus subtilis*, and *Escherichia coli*. Asperpentyn belongs to family epoxyquinone, obtained from *Curvularia* sp. G6-32 is an endophyte of the medicinal plant *Sapindus saponaria*. This compound has attractive structural complexity, functional groups diversity, and a wide range of biological activities like specific enzyme inhibitory activity (Polli et al., 2021). When *Curvularia lunata* was grown on malt extract broth in laboratory conditions, it produced three perylenequinones named methylated 12-epi-stemphytriol, alterperylenol, and dihydroalterperylenol. These compounds show potent phytotoxicity (Cruz et al., 2020). A new compound, *Curvularia hawadride* along with five known compounds have been isolated from the leaves of *Dactyloctenium aegyptium* (crowfoot grass) which shows inhibitory activity of nitric oxide (NO) production with IC_50_ values of 53.7 (Suthiphasilp et al., 2021). The chemical structures of quinones derived from *Curvularia* spp. are given in Figure 3.

### Alkaloids

**Figure 4:**
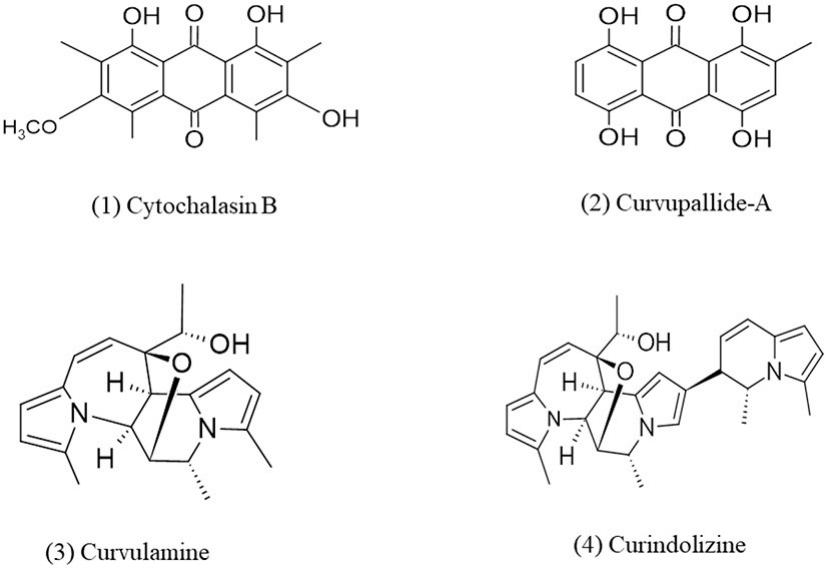
Chemical structures of bioactive compounds of alkaloid family of *Curvularia* spp. (1) Cytochalasin B, (2) Curvupallide-A, (3) Curvulamine, and (4) Curindolizine.

Alkaloids are an important group of nitrogen-containing secondary metabolites that consist of one or more nitrogen atoms within their heterocyclic ring. Biosynthesis of alkaloids can be understood by the analysis of indole alkaloid biosynthesis. Alkaloids have several biological activities such as anti-inflammatory, phytotoxicity, etc. (Debnath et al., 2018). In a study, *Curvularia pallescens* DSM 62482 were grown nitrogen-deficient condition and as a result, it synthesized some secondary metabolites which were identified as spirostaphylotrichins U and V but these metabolites did not show any phytotoxicity (Abraham et al., 1995). Addition of pyrrole alkaloid into the cell of fungus *Curvularia* sp. IFB-Z10, associated with white croaker, induces the generation of an anti-inflammatory metabolite named Curindolizine (Han et al., 2016). *Curvularia lunata* (ATCC 34690) isolated from *Litchi chinensis* Sonn. has been identified as a good source of Cytochalasin B which shows toxicity and caused abnormal coleoptile segment distortion in wheat (Wells et al., 1981). The effect of Cytochalasin B on human lymphocytes was observed earlier by Smith et al. (1967) which revealed that Cytochalasin B blocks cytoplasmic cleavage, produced extrusion of the nucleus, inhibits cell mortality, and produced multinucleated cells in treated cultures. Anti-bacterial alkaloids such as curvulamine and curindolizine which contains units of multiple electron-rich pyrrole were isolated from fungi *Curvularia* sp. and *Bipolaris maydis* and a 14-step synthesis protocol was developed through an abiotic coupling approach for their enhanced production (Xuan et al., 2021). An endophytic fungi *Curvularia verruculosa* was isolated from the leaves of *Catharanthus roseus*, this fungus has been found in the production of Vinblastine, an alkaloid that is a potent anti-cancer compound, significant cytotoxicity has been observed against HeLa cells (Parthasarathy et al., 2020). Four strains of endophytes named *Curvularia* sp. *Choanephora infundibulifera, Aspergillus japonicas*, and *Pseudomonas* sp. have been isolated from *Catharanthus roseus* cultivar Dhawal, among them *Curvularia* sp. and *Choanephora infundibulifera* showed increase production of serpentine content by 211.7–337.6% whereas *Aspergillus japonicus* and *Pseudomonas* sp. enhance the production of ajmalicine by 123.4–203.8% by upregulation of gene expression of tryptophan decarboxylase, geraniol 10-hydroxylase, and strictosidine synthase which are involved in the biosynthetic pathway of terpenoid indole alkaloid (TIA) (Singh et al., 2020). It is also observed in a similar plant that inoculation of *Curvularia* sp. (CATDLF5) and *Bacillus pumilis* (CATPS2) in the form of consortium, increased the growth of plant leaves as well as alkaloids such as vindoline (Singh et al., 2021; Figure 4).

**Figure 5:**
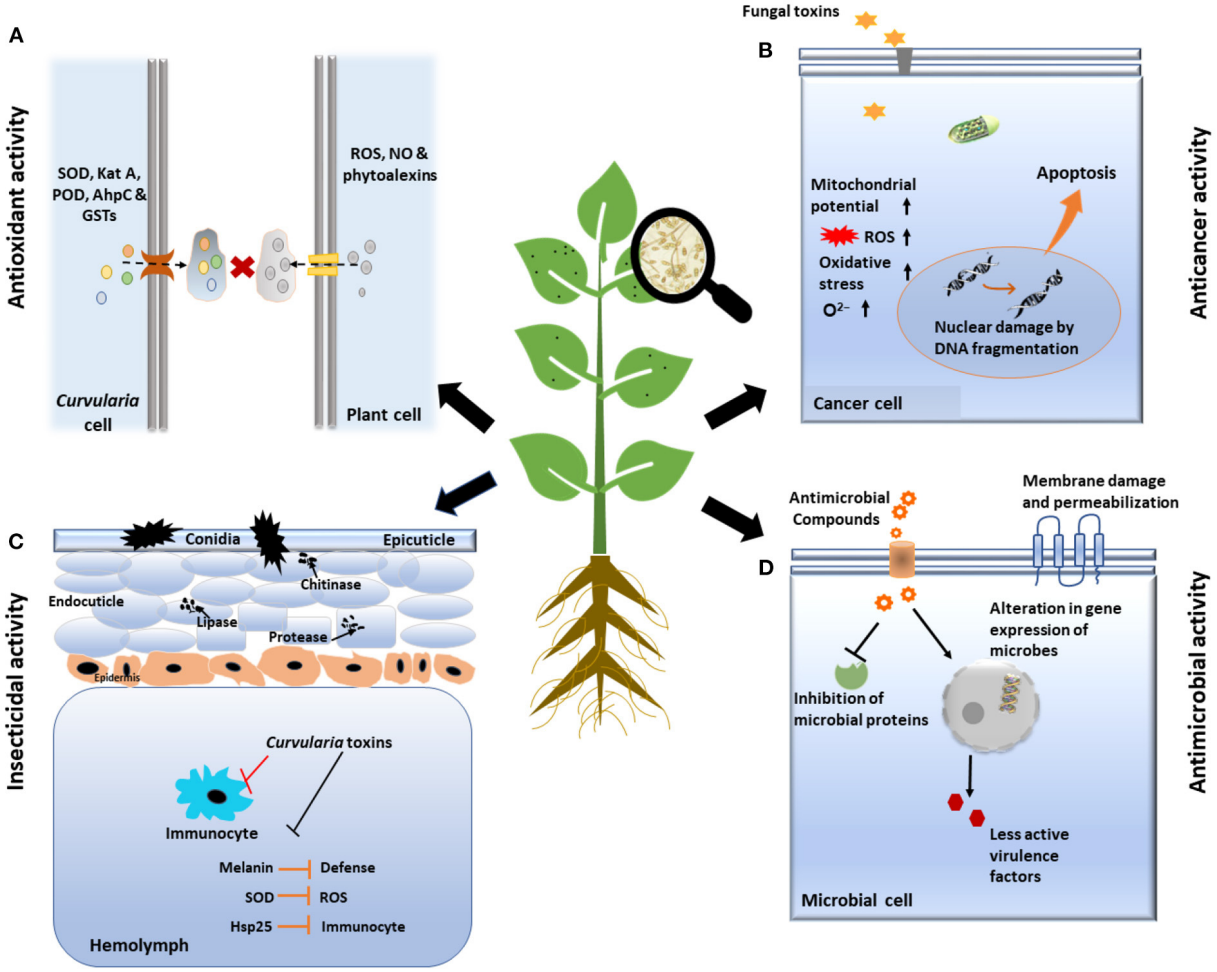
Schematic representation of various biological activities of *Curvularia* spp. **(A)** Anti-oxidant activity: Fungus secreted many anti-oxidative enzymes such as superoxide dismutase (SOD), catalase, peroxidase, etc. which counteracts to reactive oxygen species (ROS), generated by immune system of host plant. **(B)** Anti-cancer activity: Toxin secreted by fungus induced program cell death/apoptosis of cancerous cell by various processes like changing the membrane potential of mitochondria, DNA fragmentation, etc. **(C)** Insecticidal activity: *Curvularia* toxins suppress the function of insect immunocytes and inhibits immune system of insects by producing virulence factors like Melanin, Hsp25, etc. Finally, it increases level of enzymes like lipase, chitinase, protease which ultimately cause death of insect cells. **(D)** Anti-microbial activity: *Curvularia* spp. Secrets some of anti-microbial compounds, that shows antagonism against microbes by damage and permeabilization of membrane, inhibition of microbial proteins and by altering the gene expression of microbes.

The above description revealed that there are diverse metabolites synthesized and produced by *Curvularia* spp. which have several biological activities including phytotoxicity, anti-inflammatory, anti-cancer, anti-microbial, and anti-oxidant activities that are elucidated in following Table 1 and Figure 5.

**Table 1:** Various sources and biological activities of bioactive compounds of *Curvularia* spp.

**Fungi**	**Source**	**Bioactive compound**	**Nature of bioactive compound**	**Biological activities**	**References**
*Curvularia* sp. G6-32	*Sapindus Saponaria* L.	(-)-Asperpentyn	Epoxyquinone	Anti-oxidant and butyrylcholinesterase enzyme inhibitory activity	Polli et al., 2021
*Curvularia tsudae*	*Cynodon dactylon* (L.) *Pers*.	Coumarins	Phenoles	Anti-microbial and anti-oxidant activity	Nischitha et al., 2020
*Curvularia crepinii* QTYC-1	Gut of *Pantala flavescens*	Macrolide, *O*-demethylated-zeaenol, zeaenol adenosine, and ergosta-5,7,22-trien-3b-ol	Polyketides	Herbicidal and antifungal activity	Yin et al., 2018
*Curvularia papendorfii*	*Vernonia amygdalina*	Polyhydroxyacid (kheiric acid)		Anti-viral, anti-bacterial, and anti-proliferative activities	Khiralla et al., 2020
*Curvularia* sp. strain M_12_	*Murraya koenigi*	Murranofuran A, murranolide A, murranopyrone, and murranoic acid A	Polyketides	Inhibits motility against zoospores of *Phytophthora capsici*	Mondol et al., 2017
*Curvularia* sp.	Leaves of *Terminalia laxiflora*	*N*-Acetylphenylalanine, dipeptide *N*-acetylphenylalanyl-L-phenylalanine and tripeptide *N*-acetylphenylalanyl-L-phenylalanyl-L-leucine	Bioactive peptides	Suppression of tumor growth and angiogenesis by NF-κB inhibition	Tawfike et al., 2018
*Curvularia* sp.	*Gracilaria folifera*	Curvulone A and curvulone B	Polyketides	Antagonistic activity against microbes	Dai et al., 2010
*Curvularia* sp. FH01	Gut of *Atractomorpha sinensis*	Phthalic acid butyl isobutyl ester and radicinin	Polyketides	Phytotoxic activity against the radical growth of *Echinochloa crusgalli*	Zhang et al., 2011
*Curvularia trifolii*	Biological material from the veracruz reef system	Mycelial extract of fungus	–	Anti-proliferative activity against tumor cell lines	Couttolenc et al., 2016
*Curvularia* sp. BCC52426	1–3 Years old bagasse of Sugarcane	(Z)-Chloromonilinic acid B, (E)-chloromonilinic acid C, 4-chlorocurvularin, and 4-chlorocurvularinic acid	Polyketides	Anti-microbial and cytotoxic activities	Bunbamrung et al., 2018
*Curvularia* sp. RJJ-5	From erythromycin contaminated sample	3-Depyranosyloxy erythromycin A, 7,12-dyhydroxy-6-deoxyerythronolide B, 2,4,6,8,10,12-hexamethyl-3,5,6,11,12,13-hexahydroxy-9-ketopentadecanoic acid and cladinose	Polyketides	Erythromycin degradation	Ren et al., 2021
*Curvularia senegalensis* (Speg.) Subram	Soil sample collected at a Brazilian region of cerrado transition	1-Hexyl-2-propylphthalate, 1- ethyl-2-heptylphthalate, 1-hexyl-2-butylphthalate, 1-heptyl-2-proylphthalate, 1- propyl-2-nonylphthalate and two positional isomers of 1-decyl-2-butane phthalate	Phthalates	In biodegradation experiments during cultivation of commercial valuable crops	Lucas et al., 2008
*Curvularia australiensis* FC2AP	Leaves of *Aegle marmelos*	Dimer of epicatechin (DoE)	Flavanoid	Anti-cervical cancer and anti-inflammatory activity in animal models	Mani et al., 2021
*Curvularia pallescens* BAFC2336	Leaves and stems of *Baccharis cordifolia*	Compounds similar to Brefeldin A and Curvularin	Lactones	Inhibits replication of herpes simplex virus type 1 (HSV-1) and vesicular stomatitis virus (VSV)	Lemos et al., 1999
*Curvularia* sp. IFB-Z10	*Argyrosomus argentatus*	Curvulamine	Alkaloid	Anti-microbial, anti-inflammatory	Han et al., 2014
	*Argyrosomus argentatus*	Curindolizine	Alkaloid	Anti-inflammatory	Han et al., 2016
	Marine environment	Depsidones (Curdepsidones B-G)	Polyketides	Anti-inflammatory activities	Ding et al., 2019
*Curvularia* sp.	Plant material of *Garcinia* sp.	Unknown	–	Anti-mycobacterial, anti-malarial, anti-viral, anti-oxidant, and cytotoxic activities	Phongpaichit et al., 2007
*Curvularia geniculata*	*Parthenium hysterophorus*	Unknown	–	Phytohormone production and phosphate solubilization	Priyadharsini and Muthukumar, 2017

## Biotechnological applications of *Curvularia* spp. and their bioactive compounds

**Figure 6:**
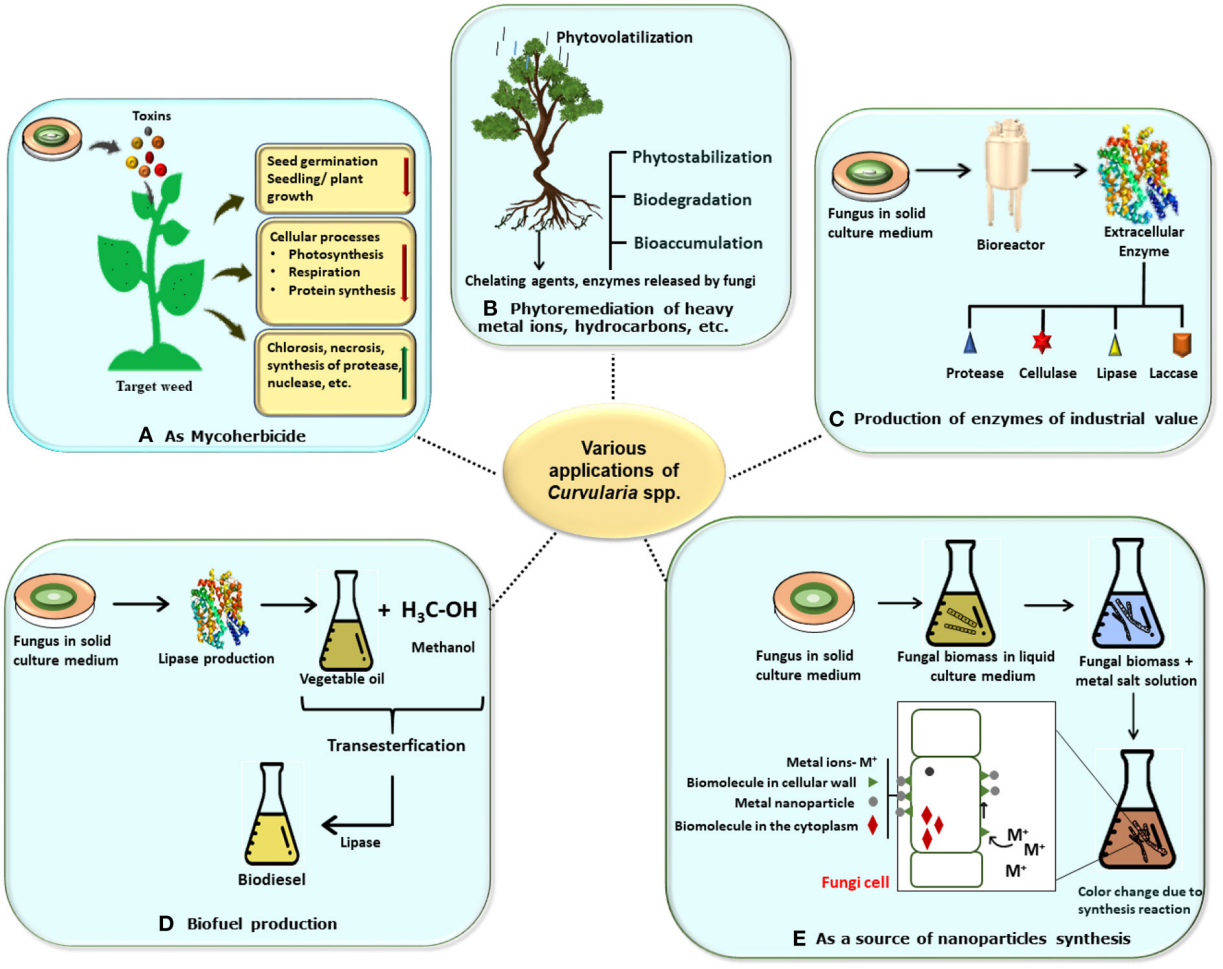
Schematic representation of various biotechnological applications of *Curvularia* spp. **(A)** Mycoherbicide: *Curvularia* spp. have great weedicidal and herbicidal capacity due to secretion of mycotoxins. Its lowers the seedling or plant growth, decrease cellular activities, and increase necrosis in host plant, thus it can be used as potent mycoherbicide. **(B)** In bioremediation: *Curvularia* spp. are a good bioremediating agents which bioremediate the plant from heavy metals and pollutants by various methods like phytovolatilization, biostabilization, biodegradation, and bioaccumulation. **(C)** In enzyme production: Large scale productions of various enzymes like lipase, laccase, cellulose, etc. which has industrial economic value can be obtained by using the fungus. **(D)** In biofuel production: Biofuel and biogas can be produce by using fungus *via* trans-esterification reaction. **(E)** As source for synthesis of nanoparticles: myconanosynthesis can be done by using fungal culture which have further several applications.

Fungi are recognized to produce a wide array of bioactive secondary metabolites such as anti-biotics, pigments, amino acids, vitamins, and organic compounds that perform various significant biological activities such as anti-microbial, anti-oxidant, anti-inflammatory, and anti-cancer and also have several biotechnological applications in the field of pharmaceutical, food, and agriculture as well as in cosmetic industries. Anti-biotics such as β-lactam, lovastatin cholesterol-lowering drug, and penicillin are some of the important products of fungal metabolism that have diverse biotechnological applications in the field of medicine and pharmaceutical industries. Apart from these, some metabolites such as tenuazonic acid, altertoxin, alternariol, alternariol monomethyl ether, thiazole, polyene, diketopiperazine, etc. have cytotoxic and phytotoxic activities (Devi et al., 2020). It is observed that plants that contain fungal species as endophytes have a capacity for nutrient acquisition, phytopathogen resistance, and ultimately plant growth (Field et al., 2015). In these respects, the commercial implementation of fungal genera is a promising tool for valuable products and their biotechnological applications are in progress (Yan et al., 2018). Poor industrialization and environmental and agricultural policies result in a vast discharge of toxic waste material such as fluorinated and chlorinated aromatic hydrocarbons, heavy metal ions, etc. So, application of the filamentous fungi and their bioactive compounds may be an alternative to these practices (Sumathi et al., 2016; Vázquez et al., 2019). The following description is concerned with various biotechnological applications of *Curvularia* spp. including fields of biotransformation, enzyme production, bioherbicides, and nanotechnology (Figure 6).

## Application in production of valuable enzymes and hormones

Many microorganisms have been identified as the main source of several economical beneficial enzymes, which have an extensive field of industrial and biotechnical applications (Adrio and Demain, 2014). They have great potential, high selectivity, high enzymatic yields, little energy costs, and easy handling conditions as compare to chemical processes (Kuhad et al., 2011). Among the all reported fungal species, members of ascomycetes including *Aspergillus niger* and *Trichoderma reesei*, basidiomycetes (*Phanerochaete chrysosporium*) and some anaerobic respiratory species such as *Orpinomyces* sp. are main source of enzymes and hormones (Fujian et al., 2001; Sindhu et al., 2016). Apart from these, some other fungi having enzymatic potential belong to the genus *Penicillium* and *Curvularia* (Banerjee and Vohra, 1991). *Curvularia kusanoi* have been identified as a potent source for cellulase, ligninase, and laccase production in grass hay and wheat straw. Experiments showed an enzymatic activity of 2,800 U/L, the specific activity of 544.74 U/g and more than 100% yield, the optimum range of the activitiy of these enzymes have been found between 30 and 40°C having high thermal stability and shows great cellulolytic and lignolytic activities (Vázquez et al., 2019). Haloperoxidase system from *Curvularia* has been found as an effective sanitizing system having great potential for disinfection of contact lenses or medical devices by facilitating the oxidation of halides, such as bromide, chloride, and iodide, to anti-microbial compounds (Hansen et al., 2003). A “B-cell” epitopes of alcohol dehydrogenase allergen have been identified from *Curvularia lunata* by immune essay and *in-silico* methods, their catalytic and binding capacity is useful to diagnose allergic diseases (Nair et al., 2011). Sharma et al. (2016) isolated extremophilic fungi from a hyper alkaline and saline lake called soda lake (Lonar lake) located in Buldhana district Maharashtra, India and identified it as *Curvularia lonarensis* sp. nov., it is also observed that this fungal species exhibited maximum phenol oxidase production for degradation of toxic phenols and lignins, so this strain of *Curvularia* fungus might be a promising and potential source of phenoloxidase. An extracellular alkaline lipase has been isolated from a novel fungus *Curvularia* sp. DHE 5 by using various substrates including wheat bran medium. Optimum temperature and pH were reported at 50°C and 8.0, respectively for this enzyme (El-Ghonemy et al., 2017). Bioefficiency of *Curvularia lunata* URM 6179 has been observed to treat textile effluent by biodegradation, this capacity is due to the production of some enzymes like laccase (Lac), lignin peroxidase (LiP), and manganese-dependent peroxidase (MnP) (Rita de Cássia et al., 2013). The application of *Curvularia clavata* in agro-industrial residues from the palm oil industry is useful for production of some lignocellulolytic enzymes such as carboxymethyl cellulase, manganese peroxidase, xylanase, lignin peroxidase, and laccase (Neoh et al., 2015). *Curvularia geniculata* mediated plant growth promotion has been reported through phosphate solubilization and phytohormone production like indole-3-acetic acid (IAA), it is also observed that production of IAA in the absence of tryptophan was 25.96 μg/ml/ whereas it was nine-fold higher in the presence of tryptophan at 232.80 μg/ml. So, this species is supposed to be a good source of mineral solubilizing agent and phytohormones (Priyadharsini and Muthukumar, 2017). *Curvularia affinis* have a great potential to produce exo- and endoglucanase from bean (*Phaseolus vulgaris* L.) biomass which is a cheap and abundant raw material, so this can be a low cost and high yield method for enzyme production (Alawlaqi and Alharbi, 2020).

## As a tool in biotransformation

Transformations of specific microorganisms play a crucial role in the syntheses of important and valuable compounds including steroids, drugs, and hormones. Filamentous fungi have great potency in the field of biotransformation. Several researches provide evidences in this context. Gene 17β-hydroxysteroid: NADP 17-oxidoreductase has been isolated from *Cochliobolus lunatus* and overexpressed in *Mycobacterium smegmatis* for transformation to enhance testosterone production (Fernández-Cabezón et al., 2017). *Curvularia lunata* has been used as a tool to understand the effects of pH and glucose concentration on the production of rifamycin oxidase in a batch reactor (Banerjee and Srivastava, 1993). Pádua et al. (2007) reported that *Cochliobolus lunatus* mediated biotransformation of digitoxigenin produces 1β-hydroxydigitoxigenin, 7β-hydroxydigitoxigenin, 8β-hydroxydigitoxigenin, and digitoxigenone as transformation products. Rifamycin B which is a clinically less active anti-biotic is transformed into Rifamycin S which is more potent and active by using rifamycine oxidase derived from *Curvularia lunata* which has a great commercial significance (Jobanputra et al., 2003). Biotransformation of corosolic acid which is an anti-diabetic agent has been carried out using *Cochliobolus lunatus*, the result of biotransformation gives rise to three new metabolites which are identified as 2α,3β,21β-trihydroxyurs-12-en-28-oic acid, 2α,3β,7β,21β-tetrahydroxy-urs-12-en-28-oic acid and 2α,3β-dihydroxy-21-oxours-12-en-28-oic acid (Feng et al., 2014). Biocatalytic structural modification in steroidal molecules of some lower eukaryotes also reported with the help of some filamentous fungi. In this mechanism, *Curvularia* sp. are capable to modify 3β-hydroxypregn-5-ene-20-one (pregnenolone) into 11α-hydroxyprogesterone, *via* changing the 3β-ol-5-ene to 3-keto-4-ene moiety (Kollerov et al., 2019). In an earlier transformation study done by Gotor et al. (2000), it was observed that when fungus *Curvularia lunata* was added to alcoholic solutions of benzoylacetonitrile. It leads diastereo- and enantioselective reactions that produce α-alkyl β-hydroxy nitriles and γ-amino alcohols in high amounts.

*Curvularia lunata* has the capacity to oxidize the 4,5-double bond to produce corresponding epoxy-ionone, in a transformation process of the isomeric forms of the damascone, apocarotenoids ionon and theaspirane (Serra and De Simeis, 2019). Biotransformation of Squamulosone [aromadendr-1(10)-en-9-one] isolated from the aromatic plant *Hyptis verticillata* Jacq. has been done with *Curvularia lunata* ATCC 12017 which results in the production of seven novel metabolites that show insecticidal activity. Meanwhile, another experiment was done with the same strain of fungus that transform 3α-hydroxycadina-4,10(15)-diene (2) to three new analogs, namely, (4S)-1α,3α-dihydroxycadin-10(15)-ene, 3α,12-dihydroxycadina-4,10(15)-diene (7), and 3α,14-dihydroxycadina-4,10(15)-diene (6) (Collins et al., 2001). A diterpene solidagenone is oxidized into 3-oxosolidagenone and 3α-hydroxysolidagenone and also gives rise to some fungal substances like as well as fungal radicinol and isoradicinol when inoculated with cultures of *Curvularia lunata* (Schmeda-Hirschmann et al., 2004). Recent study done by Yilmaz et al. (2022) suggests that *Curvularia lunata* can be a possible source of berberine transformation. Their experiments revealed that berberine concentration decreased at the end of 14 day and *C. lunata* consumed 99% of initial concentration of 0.35 mg/mL and 87% of initial concentration of 0.5 mg/mL of berberine whereas laccase and β-glucosidase enzyme activities were not much affected. Described experimental findings suggest that *Curvularia* spp. are the good transforming agent and can be applied in various fields.

## As bioremediating agent

Bioremediation is the ability of microorganisms to detoxify organic unwanted and toxic substances into non-toxic substances (Flowers et al., 1984). Filamentous fungal genera are also used in fermentation industries due to their high binding capacity with metals and nutrient concentrations and adaptability toward environmental parameters such as pH, temperature, and anaerobic conditions (Dhankhar and Hooda, 2011; Kanamarlapudi et al., 2018). Thus, fungal biomass can be used at a large scale for industrial purposes such as the removal of heavy metal ions from extremely contaminated effluents. Fungus-derived biosorbents are non-toxic and hence, they are safe and can be handled easily (Kanamarlapudi et al., 2018). Biodegradation of oil spills of petroleum hydrocarbons which are hazardous for aquatic and terrestrial ecosystems have been observed with the help of fungal isolates and their consortia. It is remarkable that *Curvularia brachyspora* has a potent ability to change polyaromatic hydrocarbons (PAHs) and saturated hydrocarbons (SH) qualitatively and quantitatively (Hamad et al., 2021). *Cochliobolus* sp. secretes various enzymes in which laccase production is useful to control plastic wastes by degradation of low molecular weight polyvinyl chloride (PVC) (Sumathi et al., 2016).

Rhizoremediation studies with fungal genera associated with *Luffa aegyptiaca* (Mill.) suggest that *Curvularia lunata* have a higher capacity to degrade spent engine oil in contaminated soil with the efficiency of 83% (Ani et al., 2021). It is also observed that when treatment is given with both plant and fungus, the degradation rate increases as compared to single inoculation. So, this study concludes that *C. lunata* increases the germination, growth, survival, and bioremediation efficacy of *Luffa aegyptiaca* in a polluted environment (Ani et al., 2021). *Cochliobolus lunatus* strain CHR4D now known as *Curvularia lunata*, has been predicted as a potential candidate for mycoremediation of high molecular weight polycyclic aromatic hydrocarbon polluted environments because it shows rapid degradation of chrysene, which is a four-ringed high molecular weight (HMW) polycyclic aromatic hydrocarbon (PAH) (Bhatt et al., 2014). *Cochliobolus lunatus* have capacity to degrade crude oil by 4.7% (Al-Nasrawi, 2012). The results of studies with *Curvularia geniculata* P1 help to understand the regulatory mechanisms of mercury tolerance and plant growth, *C. geniculata* P1 increase the host plant *Oryza sativa* L. biomass by four-fold and reduce the negative impacts of the metal on photosynthesis, several gene clusters are also involved to regulate these processes (de Siqueira et al., 2021). Pietro-Souza et al. (2020) did mercury bioremediation experiment, results revealed that *Curvularia geniculata* P1 removes 100% mercury from the cultures *via* the mechanism of volatilization and bioaccumulation.

## *Curvularia* spp. as mycoherbicide

Weed causes severe economic losses to agricultural lands and crop yield as compared to all other pests. Excessive use of chemical pesticides is hazardous for soil fertility and health and the use of eco-friendly herbicides is a good alternative in which microbes and their metabolites are points of interest nowadays. Among all microorganisms, filamentous fungi and their toxic metabolites play a key role. Due to their high toxicity to cells, they are being applied in this field and are known as mycoherbicides.

*Curvularia lunata* LD2 is a potent mycoherbicide against *Echinochloa crus-galli* (Barnyard grass) which compete with many important crops of economic value and causes serious problem in production of rice, hence is the most harmful weed species in the world. So this can be economically important tool to control the weeds in paddy fields (Nandhini et al., 2019). Both varieties of crabgrass *Digitaria sanguinalis* (L.) Scop. (Large cabgrass) and *Digitaria ischaemum* (Schreb.) Schreb. ex Muhl. (Smooth cabgrass) are invasive weeds in Canada and studies revealed that fungus *Curvularia eragrostidis* shows potent toxicity against these weeds and can be helpful to control them (Krupska and Watson, 2021). The results of the study done by Zhu and Qiang (2004) indicate that fungal isolate *Curvularia eragrostidis* QZ-2000 is a potent bioherbicide for control of large crabgrass in crop fields such as soybean, corn, cotton, peanut, and water-melon. The same fungal strain was evaluated for other plants such as *Chenopodium serotinum, Polygonum aviculare, Beckmannia syzigachna*, etc. and herbicidal characters such as inhibition of plant root elongation, reduction in chlorophyll content were determined (Shujun et al., 2006).

Curvularin and αβ-dehydrocurvularin which are potent phytotoxins have been extracted from *Curvularia intermedia*, these metabolites show severe necrotic symptoms in *Pandanus amaryllifolius*, so can be applied as mycoherbicide (Meepagala et al., 2016). O-Demethylated-zeaenol and zeaenol isolated from *Curvularia crepinii* QTYC-1 have phytotoxic activity against *Echinochloa crusgalli* and is a good herbicide and can be used as a biocontrol agent in agriculture (Yin et al., 2018). cDNA-AFLP analysis of *Digitaria sanguinalis*, after the inoculation of *Curvularia eragrostidis* showed that there is upregulation of 6 genes involved in various functions such as signal transduction, cell growth and development, energy metabolism, abscisic acid biosynthesis, and stress responses. The result of the study also showed mycoherbicidal potency of *C. eragrostidis* in this crabgrass (Wang et al., 2013). Cochliotoxin named 3-hydroxy-2-methyl-7-(3-methyloxiranyl)-2,3- dihydropyrano [4,3-β] pyran-4,5-dione, has been isolated from *Cochliobolus australiensis* a fungal pathogen which shows potent phytotoxicity against *Pennisetum ciliare or Cenchrus ciliaris*, commonly called buffelgrass, a highly invasive weed of Sonoran Desert of southern Arizona (Masi et al., 2017). Singh et al. (2021) reported mycoherbicidal potential of *Curvularia lunata* (FGCCW#21) and their metabolites against common wireweed *Sida acuta* Burm f. *Cuscuta gronovii*, a parasitic plant on black gram and lablab bean can be control by application of spore suspension of some pathogenic fungi including *Curvularia pallescens* (Bangar et al., 2019). *Xanthium strumarium* L. is an invasive weed in Sudan and responsible for various problems related to agriculture, environment, and health, *Curvularia lunata* have potent mycoherbicidal activity to control this weed by causing leaf blight (Haroun, 2015).

## As a source for myconanosynthesis

The field of nanoparticles (NPs) is emerging in current times and has several applications in various fields including medical, pharmaceuticals, pest management, crop protection, and drug delivery. NPs have been synthesized *via* chemical synthesis, physical synthesis, and green synthesis (Sharma et al., 2010; Dahoumane et al., 2016; Singh et al., 2016). Green synthesis is the biological method of NPs synthesis by using biological agents and is popular nowadays. Endophytic microorganisms such as actinomycetes, bacteria and fungi have potential to convert metal ions into metallic NPs such as Au, Ag, Cu, and Zn by secretion of cellular enzymes and their secondary metabolites (Meena et al., 2021). *Curvularia inaequalis* represents a new source for biosynthesis of silver nanoparticles, it is observed that colloidal dispersion of the silver nanoparticles has strong anti-microbial activities against *Escherichia coli* and *Klebsiella pneumoniae* (de Oliveira et al., 2013). Cell filtrate of *Curvularia pallescens* which contain many alkaloids and proteins is identified as a good candidate for the reduction of AgNO_3_ to Ag NPs, which is a highly crystalline nanoparticle, these nanoparticles exhibit great anti-fungal activity against tomato leaf mold *Cladosporium fulvum* (Elgorban et al., 2017). Fungal derived silver nanoparticles show potent anti-viral activities against human parainfluenza virus type 3 and herpes simplex virus by blocking the interaction of the virus with the cell, which depends upon the size and zeta potential of the nanoparticles. These results have been observed by application of silver nanoparticles derived from *Curvularia* spp. (Gaikwad et al., 2013). Muhsin and Hachim (2014) obtained silver nanoparticles derived from a soil fungus, *Curvularia tuberculata* and identified as a promising anti-microbial agent against pathogenic bacteria *Proteus mirabilis, Escherichia coli, Salmonella typhi, Pseudomonas aeruginosa*, and *Staphylococcus aureus* due to their broad-spectrum efficacy of inhibition. Tiny-sized (5–20 nm) spherical-shaped Nanoceria synthesized from the culture filtrate of *Curvularia lunata* exhibit anti-bacterial activity (Thakur et al., 2019).

Silver nanoparticles synthesized by filamentous fungus *Cochliobolus lunatus* show larvicidal activities against vectors: *Anopheles stephensi* and *Aedes aegypti* (Salunkhe et al., 2011a). Larvicidal activity increased with the concentration of nanoparticles. A possible site for silver absorption is supposed to be the cell wall of *C. lunatus* and some organic groups such as carboxyl, carbonyl, and secondary amines present in the cell wall of fungi have a significant role in biosorption of silver in nano form (Salunkhe et al., 2011b). Silver nanoparticle synthesized from *Curvularia affinis* Boedjin is a good nanofungicide as it inhibits the growth of the fungal pathogen *Alternaria solani* (Madhusree et al., 2018). Platinum nanoparticles (PtNPs) synthesized from *Curvularia affinis* Boedjin have significant anti-proliferative and pro-apoptotic activities in sarcoma-180 cells, and can apply as a potent anti-tumor drug (Bhattacharya et al., 2022).

## Application in thermo and drought tolerance

Global temperature is increasing day by day due to greenhouse gases emission. This climate change imposed negative impacts on abiotic as well as biotic factors of the ecosystem. Thus, flora of that area naturally develops some strategy to deal with this thermal stress. Another technique is bioengineering of plants with stress-tolerant microorganisms such as bacteria, fungi, etc. various experimental studies are going on in this area for example the thermotolerance of *Sphagnum* commonly called “Peat Moss” is modulated by the composition of its microbiome, this is due to the inductive response of heat shock proteins (HSP 70 family) evidenced by metagenomics and metatranscriptomics studies (Carrell et al., 2020). Plant fungal symbiosis is essential for thermotolerant capacity, it is observed in many studies where symbiotic *Curvularia* spp. were able to tolerate stress while others were not (Redman et al., 2002). There are many pieces of evidence available in the literature that indicates that there is a co-evolution between host and microbes for example microbiome associated with the host is able to facilitate the alleviation of stressful conditions on geothermal soils. Thermotolerant *Curvularia* increases survival up to 40°C temperature in non-adapted tomato plants, while *Curvularia* isolated from non-geothermal soils did not show such type of response (Henry et al., 2021). *Curvularia crepinii*, an endophytic fungus present in the roots of *Hedyotis diffusa* is capable to adapt according to the environment and involved in increasing thermotolerance of the host plant (Zhou et al., 2015). *Curvularia brachyspora* isolated from grasses and weedy rice plants from South Korea and the USA are reported to increase stress tolerance in host plants by increasing biomass and decreasing water consumption (Redman et al., 2021).

A double-stranded RNA virus called *Curvularia* thermal tolerance virus present within *Curvularia protuberata*, provides thermal tolerance to host plants. Expression sequence tag (ESTs) analysis suggests that there is accumulation of some osmoprotectants like glycine betaine, trehalose and taurine during heat stress response and melanin pigments and heat shock proteins of fungus are also supposed to be involved in stress tolerance (Morsy et al., 2010). *Curvularia protuberata* isolated from *Dichanthelium lanuginosum* shows cold stress tolerance in germinating seeds in laboratory conditions and heat tolerance in field conditions (Dey et al., 2019). Halophytic plant *Suaeda salsa* associated *Curvularia* sp. provides salinity stress tolerance, this is due to its plant growth-promoting attributes such as enhancement of chlorophyll a and b as well as proline contents in leaves, production of potent anti-oxidant enzymes like ascorbate peroxidase (APX) and superoxide dismutase (SOD). The fungus also protects plants by photochemical quenching (Pan et al., 2018). Endophytic fungus *Curvularia spicifera* was isolated and tested for its water stress tolerance in wheat. It was observed that the fungus improves shoot biomass and metabolism by increasing the content of proline, protein ascorbate peroxidase (APX) and K/Na ratio but the actual mechanism is unknown (Aletaha and Sinegani, 2020).

## Some other beneficial applications

The major applications of *Curvularia* spp. are described above, apart from these, there are some other applications in the field of biogas, biodiesel production, biosorption, and uranium mining. It is well-known that the cell wall of microorganisms is capable of absorbing metal ions. In an experiment, it was observed that cell debris of *Curvularia* sp. strain DFH1 degrades 85% of Cd (II) and 15% of Zn (II) in time of 1 h, when placed in a solution of artificial wastewater (Abu-Elreesh et al., 2011; Bengyella et al., 2019). Experimental findings of Di et al. (2021) also suggest that *Curvularia coatesiae* XK8 has the capacity to absorb Cd (II) and Sb (III) in polluted areas. Genus *Curvularia* represents a potential source of biodiesel production due to its capacity to produce both saturated and unsaturated fatty acids which are raw feedstock for the production of biodiesel. Cellulose degrading enzyme cellulase production observed in genus *Curvularia* and considered a source of biofuel production (Dakshayani et al., 2019). Lipids containing dry weight of mycelium have been found in about 26% of *Curvularia* sp. strain DFH1, a raw source for biodiesel production (Abu-Elreesh and Abd-El-Haleem, 2014). Enzyme vanadium chloroperoxidase derived from *Curvularia inaequalis* is useful in the production of third-generation biosensors and biofuel cells (de Almeida, 2020).

*Curvularia lunata* is an endophytic fungus of *Ficus religiosa* is a source of unsaturated hydrocarbon 1-Eicosane. This compound is of economic value in the automobile industry for biofuel production (Maheshwari et al., 2018). A large number of microorganisms including bacteria and fungi have a great ability to convert high molecular weight compounds into lower mass compounds by synthesis of extracellular enzymes like cellulase and lignocellulase and carried out biogas generation (Ferdeş et al., 2020). Biogas production increases in lignocellulosic biomass by *Curvularia lunata* mediated enhancement of the methane by 39% (Yadav and Vivekanand, 2020). *Curvularia lunata* secrete laccase enzyme. When the biological optimized treatment to wheat straw was given with *C. lunata*, the biogas production increased from 449 to 533 mL/gVS and methane from 274 to 336 mL/gVS. Whereas, for pearl millet straw, the biogas production increased from 360 to 463 mL/gVS, and methane from 220 to 305 mL/gVS (Ferdeş et al., 2020). Filamentous fungi are considered a good metal leaching agents due to their capability to survive and grow at high pH, temperatures fluctuations, low nutrient environment, and wide range of tolerance to metal concentrations (Anand et al., 2006; Ruta et al., 2010; Puglisi et al., 2012). Studies reported that *Curvularia clavata* carried out the leaching mechanism of uranium indirectly by combining uranium (${\text{UO}}_{2}^{2+}$) with oxygen (O_2_), iron (Fe^2+^), and hydronium ion (H_3_O^+^). This is differing from the direct mechanism of metal leaching where activated uranium ore (${\text{UO}}_{2}^{+}$) is formed by the combination of oxygen (O_2_) and hydrogen ions (2H^+^) with uranium ore (UO_2_) which results in 50% metal recovery from the uranium ore (Mishra et al., 2009). The *C. clavata* strain UC1FMGY isolated from the Jaduguda mine in India shows not only acidophilic activity but also produced uranium extraction with the efficiency of 50% (Mishra et al., 2009).

## Conclusion and future perspective

The use of microorganisms and their bioactive compounds in the field of biotechnology to increase the economy at a low cost and by the eco-friendly methods is popular nowadays. Filamentous fungi play a key role in these processes. There are several secondary metabolites that are known secreted by fungi. There are several types of bioactive secondary metabolites that have been synthesized from *Curvularia* spp. with various biological activities that provide benefits for the survival of fungus and hosts in harsh conditions. The studies revealed that these compounds have many beneficial applications in the field of biotechnology and are a useful tool for valuable substance production at large scale. The present article summarized metabolic diversity, its activities, and applications of *Curvularia* genus. Further studies are required to understand the genetics and molecular alteration behind the synthesis and activities of these bioactive compounds.

## Author contributions

TM and MM contributed to all chapters and had a key role in formulating the review, wrote review, critically read, and corrected the entire manuscript. TM adjusted it to the required format. TM, MM, and AN prepared the figures and the list of references. MM supervised and approved the final version of the manuscript. All the authors read and approved it for publication.
